# SD-UNet: Stripping down U-Net for Segmentation of Biomedical Images on Platforms with Low Computational Budgets

**DOI:** 10.3390/diagnostics10020110

**Published:** 2020-02-18

**Authors:** Pius Kwao Gadosey, Yujian Li, Enock Adjei Agyekum, Ting Zhang, Zhaoying Liu, Peter T. Yamak, Firdaous Essaf

**Affiliations:** 1 Faculty of Information Technology, Beijing University of Technology, Beijing 100124, China; zhangting@bjut.edu.cn (T.Z.); zhaoying.liu@bjut.edu.cn (Z.L.); peteryamak@emails.bjut.edu.cn (P.T.Y.); firdaous.essaf@emails.bjut.edu.cn (F.E.); 2 School of Artificial Intelligence, Guilin University of Electronic Technology, Guilin 541004, China; liyujian@guet.edu.cn; 3 College of Life Science and Bioengineering, Beijing University of Technology, Beijing 100124, China; enockagyekum3@emails.bjut.edu.cn

**Keywords:** biomedical image segmentation, depthwise separable convolutions, group normalization, weight standardization, computer vision

## Abstract

During image segmentation tasks in computer vision, achieving high accuracy performance while requiring fewer computations and faster inference is a big challenge. This is especially important in medical imaging tasks but one metric is usually compromised for the other. To address this problem, this paper presents an extremely fast, small and computationally effective deep neural network called Stripped-Down UNet (SD-UNet), designed for the segmentation of biomedical data on devices with limited computational resources. By making use of depthwise separable convolutions in the entire network, we design a lightweight deep convolutional neural network architecture inspired by the widely adapted U-Net model. In order to recover the expected performance degradation in the process, we introduce a weight standardization algorithm with the group normalization method. We demonstrate that SD-UNet has three major advantages including: (i) smaller model size (23x smaller than U-Net); (ii) 8x fewer parameters; and (iii) faster inference time with a computational complexity lower than 8M floating point operations (FLOPs). Experiments on the benchmark dataset of the Internatioanl Symposium on Biomedical Imaging (ISBI) challenge for segmentation of neuronal structures in electron microscopic (EM) stacks and the Medical Segmentation Decathlon (MSD) challenge brain tumor segmentation (BRATs) dataset show that the proposed model achieves comparable and sometimes better results compared to the current state-of-the-art.

## 1. Introduction

Biomedical image segmentation is the process of identifying important image components and it is a basic task in biomedical image processing which provides the basis for further and other image processing in a variety of clinical applications [1]. Some of these applications include the segmentation and quantification of gray and white matter tissues from magnetic resonance imaging brain scans for identifying various neurological diseases [2]. It usually employs partitioning a set of image pixels into subsets where the pixels in each subset are related [3]. Identifying vital information about the shapes and volumes of biological organs is very necessary and one of the most difficult tasks in biomedical image analysis [4]. In the past few years, convolutional neural networks (CNNs) have been successfully used in completing various computer vision tasks such as image classification [5,6,7], object detection [8], segmentation [9,10,11,12], action recognition [13,14], and tracking [15,16]. After outperforming state-of-the-art in image classification, researchers started paying attention to applying CNNs in structured prediction problems such as pose estimation [15] and semantic segmentation. Semantic segmentation [10,11,17,18,19] has become a major area of interest for researchers from multiple disciplines working on various types of images from biomedical to outdoor scene datasets. Automated segmentation of biomedical images could be difficult when there are large shape and size variations of the anatomy between patients as well as low contrast to surrounding tissues [20]. However, there is a rising need for automatic segmentation of medical images as a result of the complexity of manually segmenting them and recent advances have led to easier segmentation using CNNs [9,21]. One of the most significant contributions to biomedical image segmentation with CNNs is the U-Net architecture [9]. The U-Net model is very popular in biomedical image segmentation due to its ability to segment images efficiently with a very limited amount of labeled training data. Variants of U-Net have also been successfully implemented in various kinds of vision tasks. U-Net has been used with pixel-wise regression and applied to pansharpening [22]. TernausNet [23] initializes the encoder path of the architecture with weights obtained from a VGG11 [7] model pretrained on ImageNet [24] data. Attention U-Net [25] extends the standard U-Net with a proposed attention gate (AG) model for medical imaging that automatically learns to focus on target structures of varying shapes and sizes.

In recent times, there has been an increased need to implement deep learning solutions on mobile handheld devices, embedded systems or any computer with low computational budgets. A major reason why this is a challenging feat is the fact that CNNs are over-parameterized [26] and they usually require larger computing power and storage capacity for training and inference. Deep learning researchers have proposed several techniques that require pruning or quantization of weights of models pretrained on large image datasets [27,28,29,30]. Others have focused on training compact models from scratch [31,32,33] by factorizing standard convolution layers into depthwise separable convolution layers for cheaper computations.

This paper presents a similar technique used in these compact architectures also known as mobilenet architectures with the goal of training the U-Net model with fewer parameters requiring smaller storage space, less computational requirements, and faster inference. However, depthwise separable convolutions are known to have degraded performance in terms of accuracy compared to standard convolution layers. Weight standardization combined with group normalization is therefore implemented on weights of each input layer to recover its accuracy loss. This new architecture is referred to as the SD-UNet. The performance of SD-UNet is evaluated on the ISBI challenge dataset for the segmentation of neuronal structures in EM stacks and further demonstrates its robustness on brain tumor segmentation tasks on the Medical Segmentation Decathlon (MSD) challenge brain tumor segmentation dataset.

### 1.1. Motivation

There have been major shifts in technology over the past decade and the most significant of them is the migration from desktop or laptop computers to mobile and handheld devices. This means that people are naturally leaning towards deep learning solutions using their mobile devices. There is a need to develop applications that require less memory storage and low computation and battery power. Latency usually comes about as a result of time for transferring data over networks and the number of computations required by the deep learning model. Performing tasks that require low latency like timely identification and segmentation of biomedical images require data to be immediately available. Most companies and researchers currently rely on retrieving data stored on a network server or distributed on other devices usually leading to huge overhead costs especially during deployment. This also makes it difficult to continuously update training data in order to improve the efficiency of the deep neural network. The energy required by deep CNNs usually exceeds the limited on-chip memory of mobile and handheld devices, so they are sometimes supplemented with off-chip memory, which consumes a significant amount of energy. To overcome such limitations, we introduce a new variant of the U-Net architecture, the SD-UNet for efficient segmentation of biomedical images on devices with low computational budgets.

### 1.2. Contributions

The contributions of this paper can be summarized as follows:

- We propose the use of depthwise separable convolution layers to replace all standard CNN layers except the first CNN layer in the original U-Net model
- Depthwise separable convolution layers are known to achieve lower performance compared to standard convolution layers. We demonstrate that performance drop due to the process can be recovered with a method of weight standardization and group normalization.
- SD-UNet model has 8x fewer parameters and requires 23x less storage space. The computational complexity or number of floating point operations (FLOPs) required by SD-UNet is 8x less than is required by the original U-Net model and shows great performance on the segmentation of biomedical images.

The rest of this paper is organized as follows. Section 2 summarizes the background and relevant related work. Section 3 describes the materials and methods used in this study, and Section 4 presents results and discussion. A brief conclusion is finally provided in Section 5.

## 2. Related Work

In this section, we describe in detail the major previous works that motivated our work.

### 2.1. Depthwise Separable Convolutions

Depthwise separable convolutions were initially introduced by [34] and then later implemented by [31,35]. Depthwise separable convolution is a form of factorization which factorizes a standard convolution into a depthwise convolution and a pointwise convolution (1 × 1 convolution). A standard convolution layer works by applying a convolution kernel to all of the channels of the input image and takes a weighted sum of the input pixels covered by the kernel sliding across all input channels of the image. This means that for a standard convolution, no matter how many input channels are available, the output channel is one. However, in depthwise separable convolutions, features are only learned from the input channels so the output layer has the same number of channels as the input.

**Figure 1:**
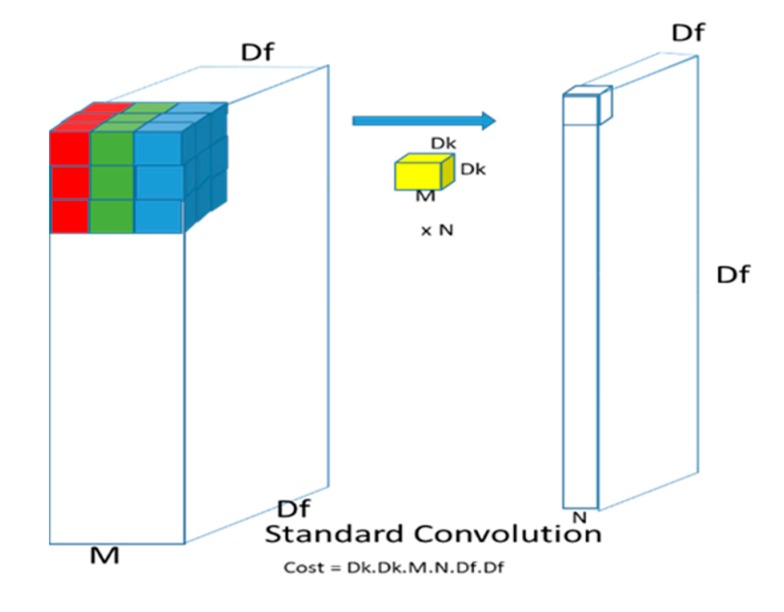
Standard Convolution.

**Figure 2:**
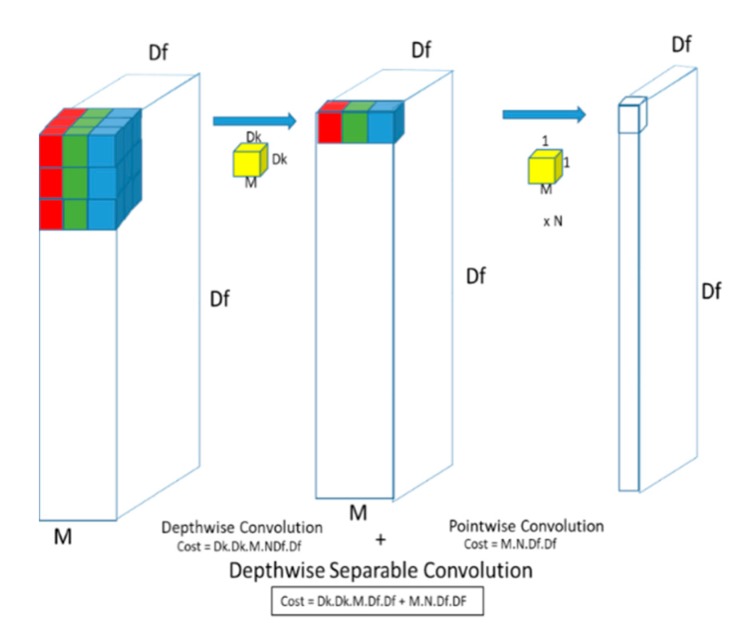
Depthwise Separable Convolution.

This is known as depthwise convolution followed by a pointwise (1 × 1) convolution layer which computes the weighted sum of all output channels into a single output (Figure 1 and Figure 2).

The cost of a standard convolution is given by:

(1) Dk×Dk×M×N×Df×Df

where *Df*, is the feature map size with *M* input channels and *Dk* is the size of the kernel with *N* output channels. The total cost of a depthwise separable convolution is also given by:

(2) Dk×Dk×M×Df×Df+M×N×Df×Df

which is the sum of the separable and the pointwise convolutions. Some deep networks [31,32,33] are able to reduce computation to 8 or 9 times as compared to standard convolutions by using 3 × 3 depthwise separable convolutions.

### 2.2. Batch and Group Normalization

**Figure 3:**
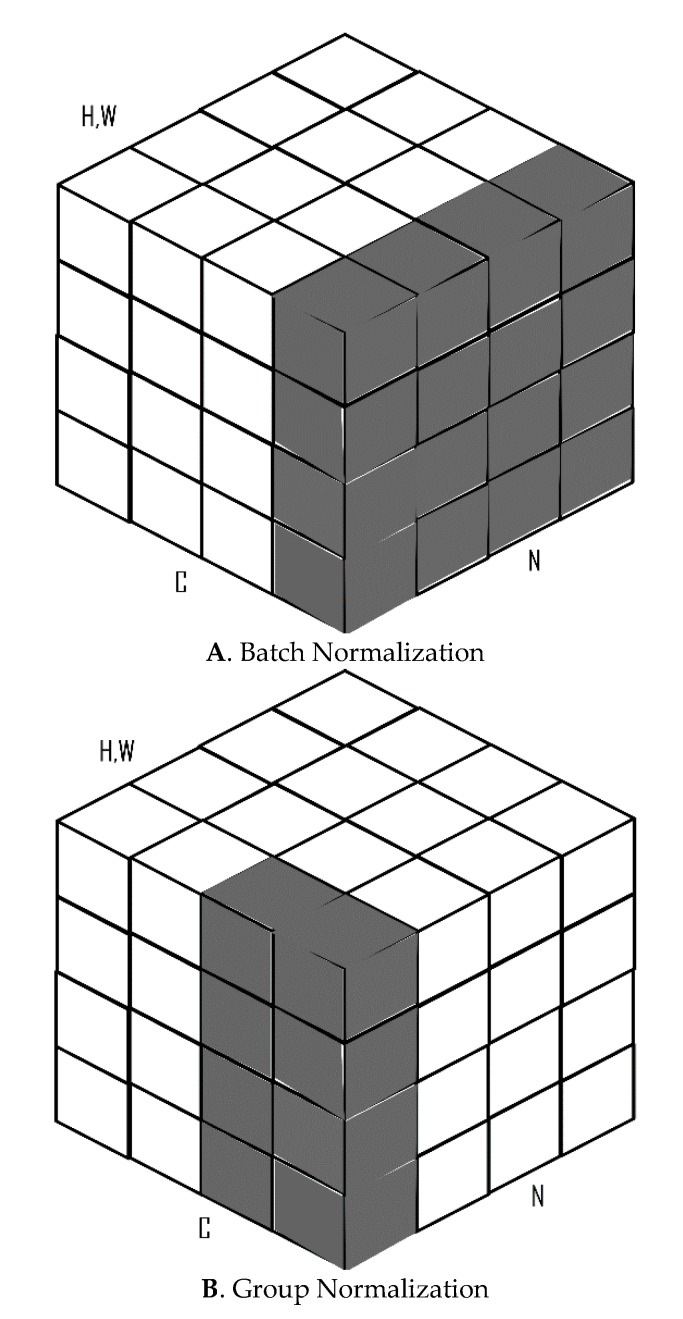
Difference between BN (**A**) and GN (**B**) as in a feature map tensor with the height and width dimensions (H, W), C as the channel axis and N as the batch axis.

Batch normalization (BN) [36] has been a widely adopted technique over the years and has proven to be very effective in several deep learning tasks. BN makes use of the mean and variance computed within a mini-batch of data to normalize its features during activations. BN standardizes activations to have zero mean and unit variance. The major advantages of BN include allowing faster convergence in fewer training iterations, providing some level of regularization, thereby reducing the generalization error. One major setback of BN, however, is that it requires significantly large batch sizes to work effectively. In applications that require high-resolution images for computations like object detection and image segmentation, BN does not work efficiently due to computational limitations. Group normalization (GN) [37] was therefore introduced as a layer that divides channels into groups and computes the mean and standard deviation over these groups of channels for each example during training (Figure 3). GN does not exploit batch dimensions. This allows it to perform better than BN with smaller mini-batch sizes (usually less than 32).

### 2.3. Weight Standardization

**Figure 4:**
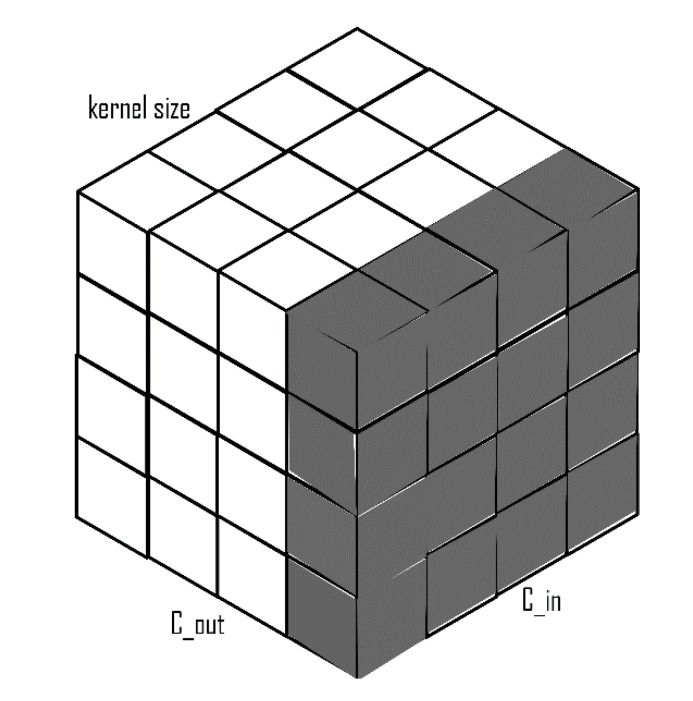
Weight standardization with C_out as the number of output channels, C_in as the number of input channels x kernel size.

Weight standardization (WS) [38] is another method of normalization which is applied to the input weights of the convolution layer, unlike BN and GN, which are implemented on the output layer or the activations (Figure 4). The main aim of WS is to standardize gradients during backpropagation. Experiments have shown that a combination of WS and GN achieves performances that are comparable to BN with large batch sizes. Given a standard convolution layer and assuming its bias term to be 0,

(3) $$y=\hat{W}*x$$

with $\hat{W}\in R^{OxI}$ as the layer weights and ∗ the convolution operation. O and I corresponds to the number of output channels and the number of input channels in the kernel region of the output channels, respectively. Instead of optimizing the loss on the original weights, $\hat{W}$ as in BN, WS represents the weights as a function of W, and optimizes the loss, L on W. So that:

(4) $$\hat{W}=WS\left(W\right)$$

Using stochastic gradient descent (SGD),

(5) $$\hat{W}=\left[{\hat{W}}_{i,j}\;|\;{\hat{W}}_{i,j}=\frac{W_{i,j}-\mu w_{i,.}}{\sigma w_{i,.}+\varepsilon }\right]$$

where $\mu w_{i,.}$ is mean of the weights, $\sigma w_{i,.}$ is the standard deviation

Therefore:

(6) $$y=\hat{W}*x$$

### 2.4. Fully Convolutional Networks (FCNs)

The most fundamental idea behind FCNs [10] is that they are only made up of locally connected layers (convolution, pooling, and upsampling) without fully connected or dense layers. This tends to reduce the time required for computation and the number of parameters. It also means that an FCN will work regardless of the input image size. FCNs are typically made up of:

- Downsampling/Contraction/Encoding Path: On this path, the model extracts and interprets the contextual information on the input image.
- Upsampling/Expanding/Decoding Path: The specific localization or construction of segmentation maps from the extracted context in the encoding path.
- Skip Connections/Bottlenecks: Combines information from encoding and decoding paths by summing feature maps

### 2.5. U-Net

The U-Net architecture is designed as an improvement of the FCN architecture specifically for the segmentation of medical images. The major difference between U-Net and FCN is U-Net is symmetrical and the bottleneck layers that combine information from the encoding and decoding paths do so by concatenating the feature maps whereas they are summed in the FCN architecture. The encoding path of U-Net is made of four blocks each containing two 3 × 3 unpadded convolutions with a ReLu activation layer and a 2 × 2 max-pooling layer. The number of feature channels is also doubled after each downsampling step but the size of feature maps is reduced due to max-pooling. The decoding path contains 2 × 2 upsampling with 3 × 3 standard convolutions. Each convolution is followed by a concatenation of features from corresponding layers in the encoding path. This helps to transfer the localization information that is learned during downsampling from the encoding to the decoding path.

## 3. Materials and Methods

In this section, we outline the proposed technique, describe the SD-UNet architecture and experiments conducted.

### 3.1. WS with Depthwise Separable Convolutions

WS has been proposed to be implemented on the weights of standard convolutions. In this study, in order to reduce the number of parameters and required computations in the U-Net model, the standard convolution layers are replaced with depthwise separable layers. WS is now implemented on the weights of the depthwise (3 × 3) convolution layers only so that,

(7) $$\hat{W}=WS\left(W_{dw}\right)$$

where $W_{dw}$ = weights of the depthwise layer and,

(8) $$y=\hat{W}*x$$

WS achieves a better and smoother loss curve during training [38] and also helps improve model accuracy as shown in Figure 10 and Table 3.

### 3.2. SD-UNet (Proposed Architecture)

**Figure 5:**
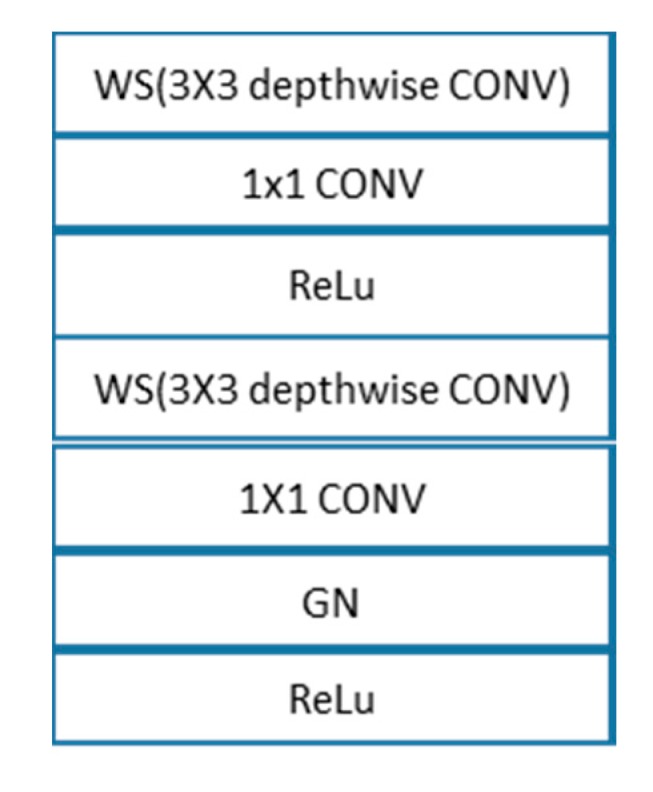
Components of one SD-UNet Block.

SD-UNet follows a similar architecture as U-Net with a few modifications. Except for the first convolution layer which has a standard convolution, all other convolution layers are made of depthwise separable convolution layers. The encoding is made up of 5 blocks:

- Block1: A standard convolution layer, a ReLu activation function, and a GN layer
- Block2 and Block3: One SD-UNet block and a max-pooling layer. An SD-UNet block is made up of two depthwise separable convolution layers, two activation layers, and one GN layer (Figure 5).
- Block4: One SD-UNet block, a dropout layer to introduce regularization [39], and a max-pooling layer. All depthwise (3 × 3) convolution layers are weight standardized.
- Block5: A final depthwise separable layer with a dropout layer.

**Figure 6:**
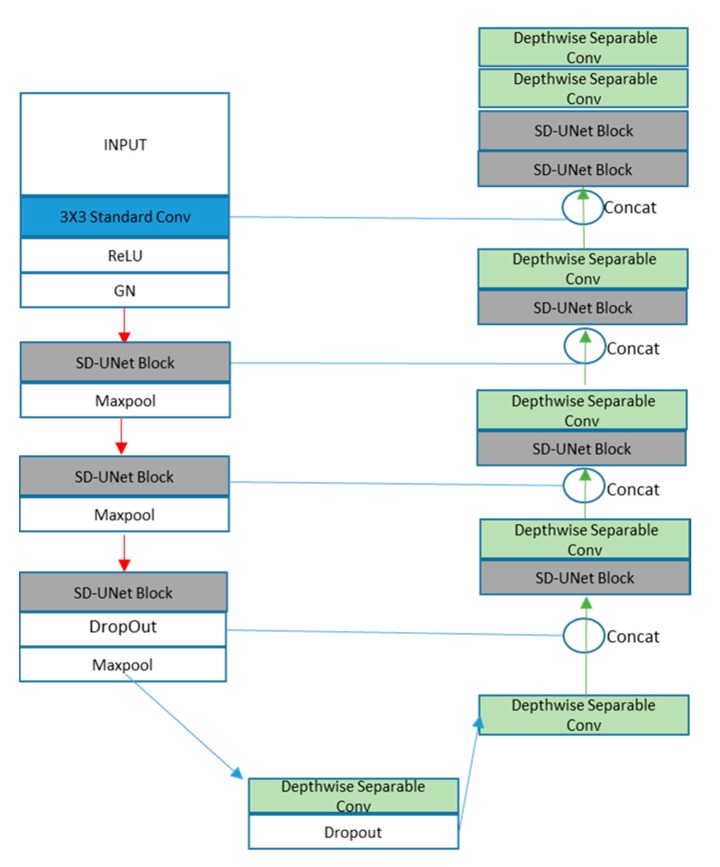
SD-UNet Architecture.

Upsampling is performed on the decoding path with a size of 2 in order to recover the size of the segmentation map. The decoding path of SD-UNet is made of a mixture of depthwise separable convolutions and SD-UNet blocks. It also consists of 5 Blocks:

- Block1: A depthwise separable convolution layer with its features concatenated with the dropout layer from Block4 of the encoding path.
- Blocks 2, 3, 4: An SD-UNet block and a depthwise separable layer concatenated with corresponding blocks from the encoding path
- Block 5: Two SD-UNet blocks and two depthwise separable layers with the last one as the final prediction layer (Figure 6).

### 3.3. Setup

The training was based on the Keras with a Tensorflow backend as the deep learning framework on a work station enabled with an NVidia Tesla K40c GPU (12GB memory) and Intel ® Xeon (R) CPU E5-2603 V4 @ 1.70 GHz with 12CPUs. CuDNN 7.0 library was used with the benchmark function enabled to ensure that the fastest algorithms are used.

### 3.4. Datasets

The datasets used to evaluate the performance of SD-UNet are the ISBI challenge dataset for the segmentation of neuronal structures in electron microscopic (EM) stacks [40,41] and the MSD challenge brain tumor segmentation dataset [42].

#### 3.4.1. ISBI Challenge Dataset

**Figure 7:**
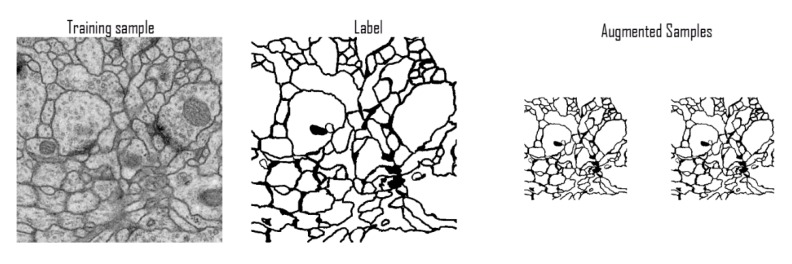
Samples of ISBI training data.

The training data is a set of 30 sections from a serial section transmission electron microscopy (ssTEM) data set of the Drosophila first instar larva ventral nerve cord (VNC). The microcube measures 2 × 2 × 1.5 microns approx., with a resolution of 4 × 4 × 50 nm/pixel. The corresponding binary labels are provided in an in-out fashion, i.e., white for the pixels of segmented objects and black for the rest of the pixels (which correspond mostly to the membranes) (Figure 7).

#### 3.4.2. MSD Challenge Brain Tumor Segmentation (BRATs) Dataset

**Figure 8:**
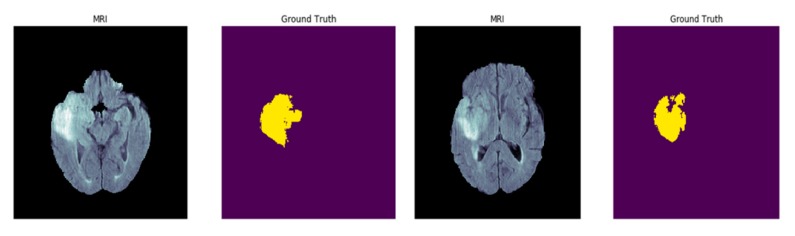
Sample magnetic resonance imaging (MRI) images and their ground truth labels from the Brain Tumor Segmentation (BRATs) dataset.

The Medical Segmentation Decathlon (MDS) Dataset is a challenge that contains 10 large datasets for medical image segmentation. In our experiments, the Brain Tumor Segmentation (BRATs) subset of the dataset is used to evaluate and compare the performance of SD-UNet. This dataset contains a subset of data obtained from BRATs challenge datasets of 2016 and 2017 [43,44,45]. Multiparametric magnetic resonance imaging (MRI) scans from 750 patients diagnosed with either glioblastoma or lower-grade glioma were also added. The MRI sequences include volumes of native (T1) and post-Gadolinium (Figure 8).

(Gd) contrast T1-weighted (T1-Gd), native T2-weighted (T2), and T2 fluid attenuated inversion recovery (T2-FLAIR) as the input channels (modality) collected for segmenting sub-regions of brain tumors which include the edema (swelling around the tumor), enhancing (Gadolinium contrast-enhanced regions), and non-enhancing (not enhanced by Gadolinium contrast) tumors with a background (no tumor) as the output channels (labels) during training.

#### 3.4.3. Data Pre-Processing

The resolution of the images of the ISBI challenge EM stacks is originally 512 × 512 but was resized to 256 × 256 due to computational limitations. Data augmentation techniques were used due to the small number of available training images. A smaller number of images might lead to a concept known as overfitting where a trained model performs very well on training data but performs poorly on new test data. These augmentation techniques included horizontal flip, zoom range, height and width shift range. The number of images of the EM stacks dataset after augmentation increased to 120. The resolution of images in the BRATs dataset (240 × 240) was also reduced to 144 × 144. Center cropping and normalization of data to ensure 0 mean and unit variance was also employed and the original 3D slices converted to 2D slices for training and testing of SD-UNet. In all, there are 75,020 MRI image samples. For training and testing, we split the images into 62,930 training, 4960 for validation, and 7130 for testing.

### 3.5. Optimization

The Adam [46] optimization algorithm was used to train the network with a learning rate of 0.0001 and 0.00001 on the EM stacks and BRATs data respectively. The loss used in training on the EM stacks dataset was based on binary cross-entropy loss. On the BRATs dataset, the loss was a weighted sum of negative dice loss and binary cross-entropy loss algorithms.

### 3.6. Performance Metrics

In this section, the major performance metrics used in evaluating the performance of SD-UNet on the datasets are explained in detail.

#### 3.6.1. Accuracy (AC)

Accuracy measures the percentage of correct predictions in any given image and is given by:

(9) $$AC=\frac{TP+TN}{TP+TN+FP+FN}$$

where *TP* = number of true positives, *TN* = number of true negatives, *FP* = number of false positives, *FN* = number of false negatives

#### 3.6.2. Intersection over Union (IOU)

The IOU or the Jaccard Index measures the percentage of overlap between the ground truth labels and the predicted outputs and is given by:

(10) $$IOU=\frac{GT\cap PO}{GT\cup PO}=\frac{TP}{TP+FP+FN}$$

where *GT* = ground truth labels, *PO* = predicted outputs.

#### 3.6.3. Sorensen-Dice Co-Efficient (Dice Co-Eff)

Dice Co-Eff Measures the Percentage of Repeated Overlaps between Ground Truth and Predicted Images and Is Different from Iou Which Takes Account of True Positives Only Once (Equation (10)). It is given by:

(11) $$Dice\;Co-eff=\frac{2\left|GT\cap PO\right|}{\left|GT\right|+\left|PO\right|}=\frac{2TP}{2TP+FP+FN}$$

#### 3.6.4. Maximal Foreground-Restricted Rand Score (V^Rand^)

V^Rand^ is defined with the intuition that given a predicted segmentation S and a ground truth T, two randomly chosen pixels belong to the same segment in *S* and the same segment in *T* with a certain probability [40] and is given by a weighted mean. The weighted mean is a combination of the Rand split score, which is the probability that two randomly chosen pixels are part of the same segment in *S*, given that they are of the same segment in *T* and the merge score, which is the probability that two randomly chosen pixels are part of the same segment in *T*, given that they belong to the same segment in *S.*

#### 3.6.5. Maximal Foreground-Restricted Information Theoretic Score (V^Info^)

V^Info^ is an alternative of V^Rand^ that measures similarity between predicted segmentation S and ground truth T. It is also the weighted mean of the information-theoretic split score and the information-theoretic mean score. It should be noted that V^Rand^ and V^Info^ are both the official metrics used by the ISBI challenge organizers while Dice Co-Eff is the metric used by the MSD challenge organizers with all scripts publicly available on their websites.

#### 3.6.6. Floating Point Operations Per Second (FLOPs)

FLOPs are simply a measure of the number of multiplications and additions of floating point numbers required to be performed by a computing device’s processor. Convolutional neural networks require such floating point operations and FLOPs are the standard metric used to measure them.

## 4. Results

Experiments measuring the computational requirements of SD-UNet, its inference speed, and segmentation performance on the mentioned datasets are conducted in this section.

### 4.1. Ablation Study

An extensive ablation study is performed to evaluate the performance of the proposed model and to support the final design decisions made in this study. Four different modifications are made to the architecture design and they include:

- U-Net (GN = 32)—Original U-Net architecture with GN only with 32 groups.
- U-Net (depthwise + BN)—U-Net architecture with depthwise separable layers replacing standard convolution layers and BN layers only
- U-Net (depthwise + GN)—U-Net architecture with depthwise separable layers replacing standard convolution layers and GN layers only
- SD-UNet (depthwise + BN + WS)—Proposed SD-UNet based on BN, WS, and depthwise separable convolutions

The performance of these modifications is reported alongside the original U-Net architecture, the proposed SD-UNet based on GN in Table 1, Table 2 and Table 3.

### 4.2. Computational Results

SD-UNet is measured for its computational requirements in FLOPs, storage requirements, a number of parameters, and inference speed and compared with the original U-Net model. In terms of computational complexity, SD-UNet requires approximately 8× fewer FLOPs compared to U-Net as does all other modifications that have depthwise separable convolution layers. Additionally, SD-UNet is approximately 81 milliseconds faster than U-Net in prediction speed for an input dimension of 256 × 256 × 1 on a single NVidia Tesla K40C GPU device. SD-UNet is also 23× smaller. U-Net (depthwise + GN=32) achieves the fastest inference on a single test image with 87 milliseconds but is still 3x the size of SD-UNet (Table 1).

### 4.3. Results on ISBI Challenge Dataset

**Figure 9:**
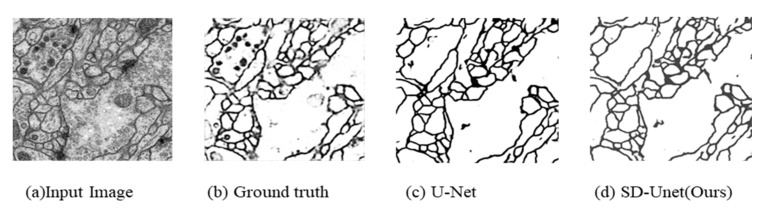
Sample Segmentation on electron microscopy dataset. (**a**) Input image; (**b**) Ground truth; (**c**) U-Net; (**d**) SD-Unet (Ours).

SD-UNet is seen to achieve comparable performance in terms of accuracy, mean IOU and Dice co-efficient, while being more computationally efficient than the original U-Net model. U-Net (GN = 32) achieves higher than all the reported models. However, it obtains the slowest prediction time and is 19× bigger in size than SD-UNet. Moreover, the difference in mean IOU and dice co-efficient is quite negligible considering tradeoffs against computational demands, storage requirements, and inference speed. Segmentation results were submitted to the ISBI challenge website and SD-UNet achieved maximal foreground-restricted Rand score after thinning: 0.914200251 and maximal foreground-restricted information theoretic score after thinning: 0.967836631 and has since been published on the available leaders’ board on the challenge website (available online: http://brainiac2.mit.edu/isbi_challenge/, accessed on 16 February 2020). A visual sample of segmentation results is shown in Figure 9.

### 4.4. Results on BRATs Dataset

**Figure 10:**
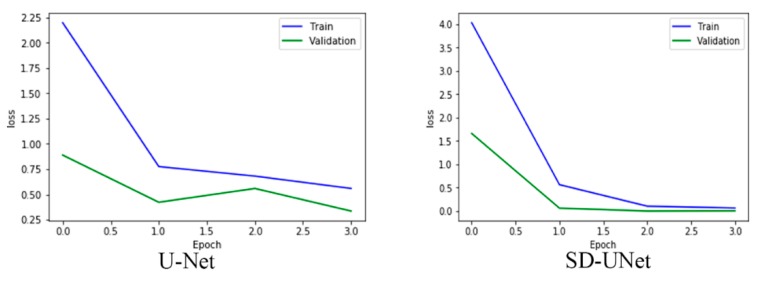
SD-UNet shows a faster convergence and improved loss during training.

**Figure 11:**
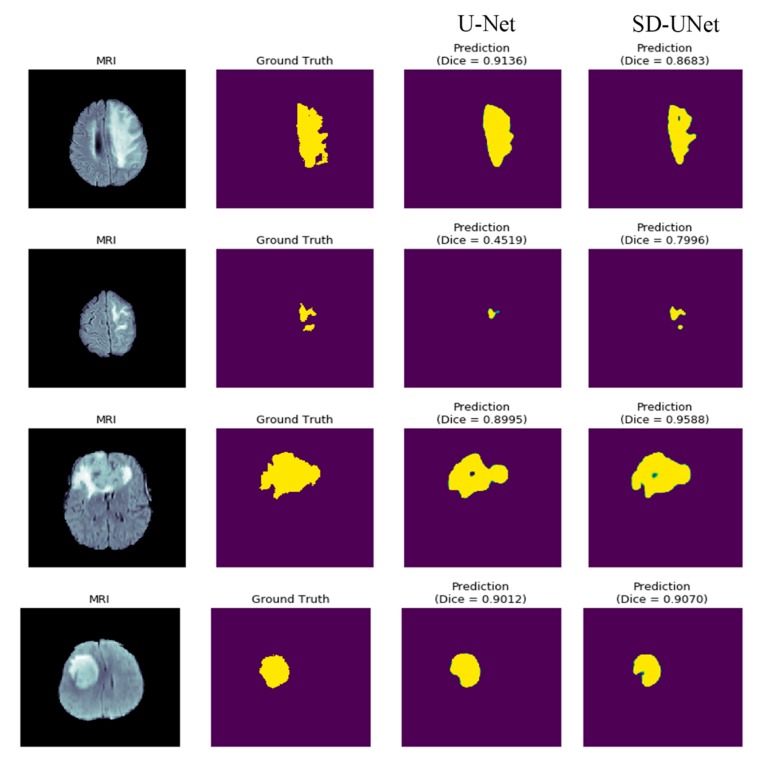
Sample Segmentation results on sample images from our test split. SD-UNet significantly performs better than U-Net on smaller tumors. The green-colored regions are simply a function of the plots that specify edges and show regions around the segmentation that are not part of the background.

U-Net and SD-UNet are trained from scratch for only four epochs and their mean dice scores on validation data compared alongside their inference speed. The choice of a smaller number of epochs is due to the ability of the Adam optimization algorithm reaching a minimum quickly and each epoch runs 1000 iterations over the dataset. SD-UNet achieves a better loss and mean dice co-efficient compared to the U-Net model. Its inference speed is also faster on a single Tesla K40C gpu device. The training curve in Figure 10 also shows that WS with GN also significantly improves the training loss and obtains a smoother curve. Pixel wise, accuracy has been accepted as a general metric but is not necessarily the best form of performance evaluation mostly due to class imbalance. This means that accuracy could be very high or very low depending on the scale of pixel imbalance that exists in the dataset and, therefore, is not necessarily always correlated with the Dice coefficient which measures the difference in the overlap between each pixel in an image and its prediction. The Dice coefficient is not dependent on the balance of data and is more accurate compared to pixel accuracy. Sample tumor segmentation visualizations are shown in Figure 11 and it is interesting to note that while SD-UNet achieves comparable performance with U-Net on large tumor segmentations, it significantly outperforms U-Net on smaller tumor segmentations.

## 5. Discussion and Conclusions

Biomedical image segmentation is an important preliminary step in the identification of tissues in image scans to aid in illness diagnosis, treatment, and general analysis. Early diagnosis is necessary to help in preventing complications that may arise due to late detections. However, with the increasing availability of large biomedical data, the workload on neurologists, radiologists, and other experts in the field has also increased. To help provide easier, accurate and timely detections, several deep learning methods have been proposed and most have chalked great successes in these tasks. The U-Net architecture is one such model that is widely accepted among researchers for biomedical image segmentation tasks.

In recent times, mobile handheld devices have been enabled with processing functionalities that were only imaginable for large computers in the past. However, deep learning applications require even higher computations. This makes it very challenging to deploy deep learning applications on handheld or embedded devices. The U-Net architecture, for instance, requires over 62M FLOPs and over 370 megabytes (Mb) of storage space which are really high demands. Moreover, not much attention has been paid to applying deep learning methods on resource-constrained devices in areas of biomedical imaging.

In this study, Stripped-Down UNet (SD-UNet), has been presented for the segmentation of biomedical data on devices with limited computational budgets. The SD-UNet architecture makes use of depthwise separable convolutions (Figure 6). However, the disadvantage of depthwise convolutions compared to standard convolutions is lower accuracy performance. It is highlighted that the problem of expected performance degradation is resolved by introducing the weight standardization algorithm with the group normalization method.

Our findings show that the proposed architecture is only 15.8 Mb in size which is 23× smaller than the U-Net and requires 8× less computational complexity (less than 8M FLOPs) (Table 1) while maintaining decent accuracy results. This means that SD-UNet can be deployed on embedded devices and any handheld device with a low computational ability such as mobile phones. Based on the results from the experiments done on the benchmark dataset of the ISBI challenge for segmentation of neuronal structures in electron microscopic (EM) stacks and the MSD challenge brain tumor segmentation (BRATs) dataset, it is seen that SD-UNet performs impressively on biomedical images. Test results on MRI scans on the BRATs dataset set show that SD-UNet achieves an average dice score of 82.75, which is in agreement with the ground truth data labeled by neuroradiologists with a dice score between 75.0 and 85.0 [45]. Additionally, SD-UNet is shown to have faster inference speed on test data and is conducive for situations where quick and accurate segmentation results are required.

Furthermore, in the absence of experts for different unforeseen reasons, being able to deploy SD-UNet on a device such as a mobile phone could help anybody in obtaining segmentation results given the availability of images. SD-UNet’s robustness is also demonstrated during test results to perform significantly better than the original UNet architecture on smaller brain tumor segmentations and can be extended to other tasks such as lung cancer detection in CT scans, skin lesions detection, breast cancer detection, and many other similar biomedical applications.

**Figure 12:**
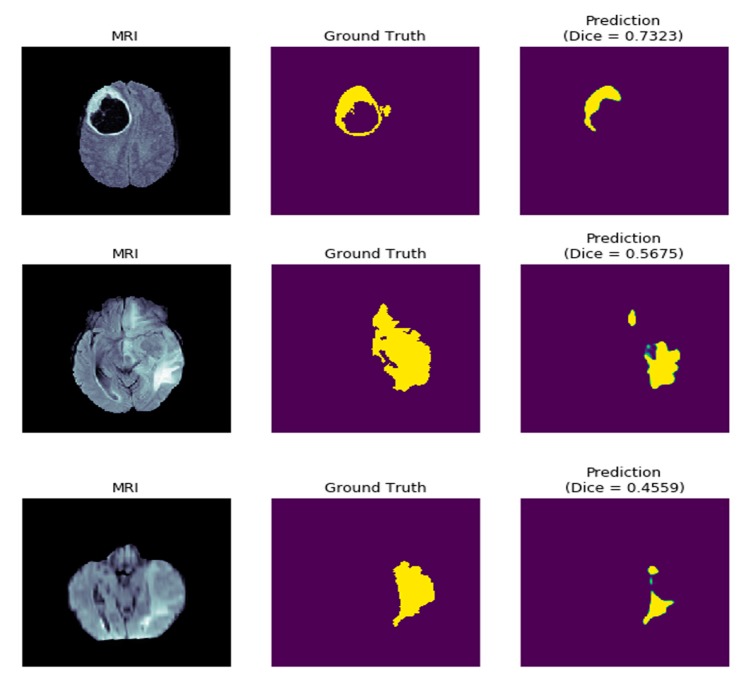
Sample SD-UNet poorly segmented tumors in the test dataset.

There are a few cases, however, where dice scores on test images fall under 75.0 (Figure 12). These may be due to factors relating to data preprocessing and hyperparameter tuning. In future work, the authors intend to continue research into designing deep architectures that require even fewer computations and target work on embedded devices as well while achieving higher test results. SD-UNet will also be applied to different kinds of biomedical data for further testing of its performance.

## Figures and Tables

**Table 1:** Computational comparison of SD-UNet and other models.

Model	# Params	# Flops	Size in Memory	Inference (ms)
U-Net	31M	62.04M	372.5MB	188
U-Net (GN = 32)	26M	51.9M	311.7MB	283
U-Net (depthwise + BN)	**3.9M**	7.8M	47.0MB	94
U-Net (depthwise + GN = 32)	**3.9M**	**7.7M**	47.1MB	**87**
SD-UNet (depthwise +BN + WS)	**3.9M**	7.8M	**15.8MB**	99
SD-UNet (Proposed)	**3.9M**	7.8M	**15.8MB**	107

“#”denotes the total number of, results in bold text denote the best values for that metric column.

**Table 2:** Comparison of results on the ISBI challenge dataset.

Model	Loss	Accuracy	Mean IOU	Dice Co-Eff
U-Net	0.0533	97.67	87.35	98.51
U-Net (GN = 32)	0.0439	**98.08**	**88.67**	**98.77**
U-Net (depthwise + BN )	**0.0435**	93.62	74.62	95.91
U-Net (depthwise + GN = 32)	0.1393	93.94	76.54	96.13
SD-UNet (depthwise + BN + WS)	0.1065	96.36	82.10	97.67
SD-UNet (Proposed)	0.0775	96.73	83.26	97.84

Results in bold text denote the best value for that metric column.

**Table 3:** Performance comparison between U-Net and SD-UNet.

Model	Training Loss	Test Accuracy	Dice Co-Eff	Inference(ms)
U-Net	0.5601	**98.71**	80.30	91
SD-UNet (Proposed)	**0.0666**	98.66	**82.75**	**56**

Results in bold text denote the best values for that metric column.
